# Determination of biomarkers from microarray data using graph neural network and spectral clustering

**DOI:** 10.1038/s41598-021-03316-6

**Published:** 2021-12-13

**Authors:** Kun Yu, Weidong Xie, Linjie Wang, Shoujia Zhang, Wei Li

**Affiliations:** 1 College of Medicine and Bioinformation Engineering, Northeastern University, Shenyang, China; 2 School of Computer Science and Engineering, Northeastern University, Shenyang, China; 3 Key Laboratory of Intelligent Computing in Medical Image MIIC, Northeastern University, Ministry of Education, Shenyang, China

**Keywords:** Computational biology and bioinformatics, Biomarkers

## Abstract

In bioinformatics, the rapid development of gene sequencing technology has produced an increasing amount of microarray data. This type of data shares the typical characteristics of small sample size and high feature dimensions. Searching for biomarkers from microarray data, which expression features of various diseases, is essential for the disease classification. feature selection has therefore became fundemental for the analysis of microarray data, which designs to remove irrelevant and redundant features. There are a large number of redundant features and irrelevant features in microarray data, which severely degrade the classification effectiveness. We propose an innovative feature selection method with the goal of obtaining feature dependencies from a priori knowledge and removing redundant features using spectral clustering. In this paper, the graph structure is firstly constructed by using the gene interaction network as a priori knowledge, and then a link prediction method based on graph neural network is proposed to enhance the graph structure data. Finally, a feature selection method based on spectral clustering is proposed to determine biomarkers. The classification accuracy on DLBCL and Prostate can be improved by 10.90% and 16.22% compared to traditional methods. Link prediction provides an average classification accuracy improvement of 1.96% and 1.31%, and is up to 16.98% higher than the published method. The results show that the proposed method can have full use of a priori knowledge to effectively select disease prediction biomarkers with high classification accuracy.

## Introduction

Microarray data are used in clinical medicine by analyzing genetic differences in tissues and cells. Effective gene selection can significantly enhance the disease prediction and diagnosis process, It has also been extensively studied in cancer pathogenesis and pharmacology. In bioinformatics, generates nonlinear datasets with multi-features and high noises. Thousands of gene expression values can be simultaneously detected in one experiment by gene chip technology, which in turn generates millions of gene expression data. Likewise, a large number of protein expression profile data can be obtained from a particular set of biological samples under different conditions by protein mass-spectrometry. However, the conventional pattern recognition methods are not suitable for the data with high dimension and few samples^1^. For such data, how to remove redundant features, and mine the useful biological information hidden in the massive data has become the key to the research of recognition.

When the number of samples is limited, the computational complexity of the classification will exhibit exponential growth increase along with the addition of features. In this case, “Curse of Dimensionality” will appear. Feature selection methods can be used to solve the following problems^2^. Effective feature selection can improve the generalization performance of learning algorithm and simplify the learning model. Based on the classification problem, the classical feature selection methods are mainly divided into Filter, Wrapper, and Embedded methods according to the feature evaluation criteria^3^.

Some advanced hybrid and ensemble feature selection methods have been reported in^4–7^. However, most of these methods are based on improvements and combinatorial optimization of existing methods and rarely consider the true dependencies between features. Although Lee et al.^8^ reported the use of probabilistic graphical models to describe feature dependencies, however, this method does not introduce a prior knowledge.

In biological information, the interaction between genes and proteins has been proved to be effective^9–11^. These features are incomplete and with a lot of noise, which requires pre-processing. Dutta et al.^12,13^ introduced a protein interaction network for genetic algorithm for multivariate optimization and have achieved better results. However, the literature only uses IntScore to deal with protein dependence and does not evaluate potential feature dependence.

Researchers have proposed to use graph structure data combined with neural networks for biomarker selection with advanced results^14,15^. To further mine the information of graph structure data and to solve the above problems, we proposed a link prediction technology based on graph neural network to achieve the improvement of gene network, using spectral clustering method combined with feature selection technology to achieve the determination of biomarkers, and the experimental results proved the effectiveness and advancement of this method.

## Related work

Traditional feature selection methods are mainly divided into Filter, Wrapper and Embedded methods. Filter method usually evaluates the features according to the inherent characteristics of the dataset, which sorts all the features and only reserves an optimal subset of the original features^16^. This method usually relies on the general characters of data to evaluate and select feature subset^17^. When using this method for feature selection, each feature is regarded as independent, i.e. there is no relationship between features.

Wrapper method takes feature selection algorithm as a part of the learning algorithm, which uses classification performance as a standard to evaluate the importance of features^18^.

Some classification algorithms embed feature selection into learning algorithm, which are called Embedded method. Embedded method is different from Filter method and Wrapper method. There is a clear difference between the process of feature selection and the process of model training in Filter method and Wrapper method^19^.

In recent years, hybrid and ensemble methods have achieved better results in the feature selection of microarray data. A feature selection algorithm called Nested-GA has been proposed recently^20^. This method combines T-test with two nested genetic algorithms, one of which is used to analyze gene microarray data and the other is used to process DNA data. A two-stage classification model based on feature selection and difference representation paradigm is proposed^21^, the first stage generates a subset of best genes by ReliefF algorithm, the second stage constructs the classifier by using the different spaces formed by the selected gene. Peng et al.’s method is proposed for high-dimensional microarray data, the method combines genetic algorithm and RFE algorithm^16^. It has used two-category datasets and multi-category datasets. Ooi et al. proposes a two-stage sparse logistic regression method^22^. This method first retains the genes that are highly correlated with cancer levels by a feature selection method in the first stage. In the next stage, solve the problem that the genes selected are highly correlated in the first stage by adaptive lasso algorithm.

Genes with similar patterns of expression^23^, synthetic lethality^24^, or chemical sensitivity^25^ often have similar functions. Additionally, function tends to be shared among genes whose gene products interact physically^26^, are part of the same complex^27^, or have similar structures^28^.

Graph Neural Network GNN^29^ provides support to process non-Euclidean structure data. It has been maturely applied to social science^30,31^, protein interaction network^32^, knowledge graph^33^, and other research fields^34^. Link prediction based on graph has been widely used^35,36^, but we have not found any research that applies this technique to feature selection previously.

**Figure 1:**
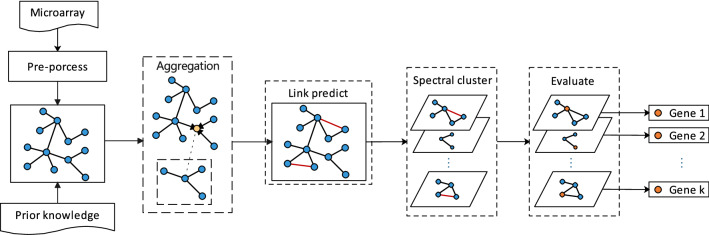
The flow of our proposed method. The aggregation process takes the first-order neighborhood of orange node as an example.

The flow of our proposed method is shown in Fig. 1. Firstly, a graph neural network is used to achieve the propagation and fusion of information from the nodes of the gene network. Link prediction techniques are used to complement the potential dependencies in the network. Subsequently spectral clustering techniques are used to divide the whole graph into sub-clusters to achieve clustering of features. Finally, a linear model is used in each subcluster to evaluate feature weights and output feature rankings.

The main contributions of our method are:

1. A gene network is used as prior knowledge in the feature selection process.
2. Proposal to enhance the feature dependencies of gene networks using a link prediction method based on graph neural networks.
3. Combining spectral clustering into feature selection for improving disease prediction accuracy.

The rest of this article is organized as follows: The Method section introduces the data sets and methods used in this article, including the establishment of graph structures, link prediction and spectral clustering. The Experiment section is the experimental part of this article. We compared traditional methods, tested the effect of link prediction, and compared advanced methods to prove the effectiveness and advancement of our method. The Conclusion section summarizes the full text.

## Methodology

### Datasets and evaluation

Microarray data can be mathematically represented as matrix $X=\left( x_{ij}\right) _{n \times d}$. Each column represents a gene and each row represents a sample for diagnosis^21^. The value of $x_{ij}$ can be expressed as the expression value of a particular gene $j\left( j=1,\dots ,d\right)$on a particular sample $i\left( i=1,\dots ,n\right)$. For a given training set $\left( x_i,y_j\right) ^n_{i=1}$ ,where $x_i=\left( x_{i,1},x_{i,2},\dots ,x_{i,d}\right)$ represents the expression value vector of the *i*-th gene, and $y_i\in \{0,1\}\left( i=1,\dots ,n\right)$ (taking the binary classification task as an example) is the sample label.

The dataset used includes DLBCL(GSE68895) and Prostate(GSE68907). DLBCL is the gene data of diffuse large B-cell lymphoma^37^. Prostate is a prostate cancer dataset^38^. Each dataset in the experiment was referenced to a corresponding GPL platform file, which allowed the conversion of probe numbers to gene names to create graph networks.

The evaluation indexes we adopted are widely used by researchers at present, which include Accuracy, Specificity, Sensitive and *Auc* values, in which *Auc* value is the area covered by *ROC* curve. In order to make the experimental results more clearly, we use *Acc* as the main evaluation. More detailed experimental results can be obtained from Supplementary Material.

### Establishment of gene relationship graph structure

We first use prior knowledge to build gene network. GeneMANIA provides a large amount of functional association data that can help us find other genes related to a set of input genes. These association data include interactions, pathways, co-expression, co-localization, and protein domain similarity^39^. In a gene network, physical interactions reflects a direct association of the functional products of genes, i.e., proteins among each other. These products often work together or even form a complex structure, which are important for carrying out biological processes. In most cases, one of these genes changes can alter or affect the activity of the other. In this study, we use physical interaction to represent a relationship between two gene candidates.

In order to apply the information data provided by GeneMANIA, we first need to obtain the GEO platform data file and convert the corresponding gene probe into a gene name. The construction process of the graph structure is as follows.

Firstly, the gene microarray data can be defined as $S=\left\{ S_{1}, S_{2}, S_{3}, \ldots , S_{N}\right\}$, *N* represents the number of samples. The feature set (gene ID set) corresponding to each sample is defined as $F=\left\{ F_{1}, F_{2}, F_{3}, \ldots , F_{M}\right\}$, *M* represents the number of features. Therefore, the expression value of any sample $S_i$ on feature $F_j$ can be expressed as $X_{ij}$. Next, the physical interaction between features is obtained from GeneMANIA as the relationship matrix *R*, which contains the relationship coefficients between any known two features $F_i$ and $F_j$. Finally, use the obtained weight matrix *R* to construct a gene relationship graph $G=(V,E)$, where $V=\left\{ V_{1}, V_{2}, V_{3}, \ldots , V_{M}\right\}$, each node $V_i$ corresponds to a vector $F_i$, and the edge relationships *E* are determined by the relationship matrix *R*.

### Graph neural network message propagation (information aggregation)

**Figure 2:**
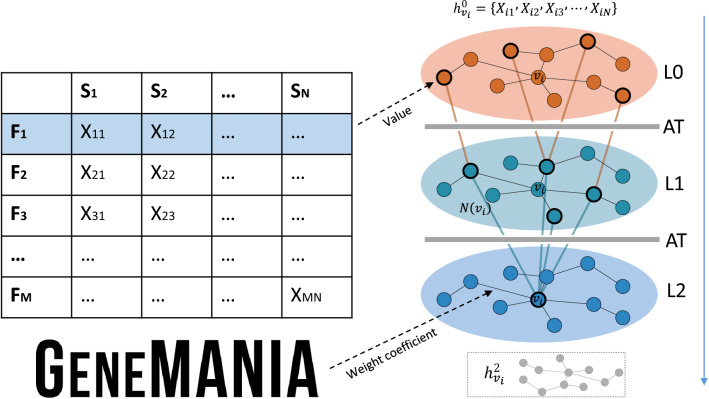
Flow chart of GNN framework visualization. The initial information of nodes is obtained from microarray data, and the edge information is obtained from GeneMANIA. *L* denotes the number of layers, and AT denotes the attention layer, which is used to process the edge weights. The figure shows a three-layer information propagation aggregation framework. node $V_i$i obtains a hidden state vector $h_{v_{i}}^{2}$ with global representation capability after continuously aggregating information from first-order neighborhood nodes.

Before link prediction, the GNN framework is first used to implement the node information propagation and aggregation operations, so that the global information representation of a single node can be used for better link prediction. The idea of message propagation and aggregation comes from GraphSAGE^40^, and we add the edge attention for processing the link weights between different nodes. The flow of this framework is shown in Fig. 2. The detailed implementation process is as follows.

Define a hidden state variable $h_{v i}^{L}$ for each node $V_i$, $L=1,2, \ldots , K, \ldots , L$ denotes the number of layers of the graph neural network. Initialize hidden state vector $h_{v i}^{0}=\left\{ X_{i 1}, X_{i 2}, X_{i 3}, \ldots , X_{i N}\right\}$ for any node. $N_{(vi)}$ is used to represent the nodes in the first-order neighborhood of $V_i$. The aggregation function shown in Eq. (1) is used to update the hidden state vector at the next level of each node.

1 $$\begin{aligned} h_{N\left( v_{i}\right) }^{K} \leftarrow \text{ AGGREGATE } _{K}\left( \left\{ h_{N\left( v_{i}\right) }^{K-1}, \forall v_{i} \in N\left( v_{i}\right) \right\} \right) \end{aligned}$$

where AGGREGATE $_{K} (*)$ represents the aggregation function of the $K-th$ layer. The strategy of averaging aggregation combined with the edge attention mechanism is used, i.e., the vector of each node belonging to the first-order neighborhood node of that node is stitched, and then each dimension is averaged and multiplied by the edge weight coefficient. The $K-th$ level hidden state vector of this node is subsequently updated using Eq. (2).

2 $$\begin{aligned} h_{v_{i}}^{K} \leftarrow \sigma \left( W^{K} \cdot {\text {COUNCAT}}\left( h_{v_{i}}^{K-1}, h_{N\left( v_{i}\right) }^{K}\right) \right) \end{aligned}$$

where $\sigma (*)$ represents the nonlinear activation function, $W^K$ represents the weight matrix of the $K-th$ layer, and COUNCAT$(*)$ represents the splicing function. Finally, Eq. (3) is used to normalize the node vector to avoid discarding too small values and to update the hidden state vector $h_{v_{i}}^{K}$ of each node.

3 $$\begin{aligned} h_{v_{i}}^{K} \leftarrow h_{v_{i}}^{K} /\left\| h_{v_{i}}^{K}\right\| _{2}, v_{i} \in v \end{aligned}$$

In the complete GNN message propagation and aggregation process, set $i=1,2,\dots ,m$,$K=1,2,\dots ,L$. Repeat the above steps to obtain the hidden state vector representation *H* of all nodes at the $L-th$ level, which is shown in Eq. (4).

4 $$\begin{aligned} H=\left\{ h_{v_{1}}^{L}, h_{v_{2}}^{L}, \ldots , h_{v_{m}}^{L}\right\} \end{aligned}$$

where *L* denotes the number of layers, $h_{v_{i}}^{L}$ denotes the $L-th$ level hidden state vector of node $V_i$. The process of node information propagation and aggregation has been completed so far, and each node $V_i$ can be considered to have a hidden state vector $h_{v_{i}}^{L}$ capable of global information representation.

### Link prediction

The link prediction process uses node hidden state vectors as node information. The purpose of link prediction is to predict the existence of edges between two nodes in the graph, which is essentially a binary classification task. Therefore, we take the edges that exist in the graph as positive samples, negatively sample some edges that do not exist in the graph as negative samples, and divide the positive and negative samples into a training set and a test set. The specific procedure is as follows.

First, it is necessary to construct positive and negative samples for training the prediction model, mark the edges that already exist in the gene relationship graph *G* as positive samples, and the set of all positive samples is called the positive sample set *Pos*.

In the process of constructing negative samples, the existing links between any pair of nodes $(v_j,v_r)$ in the gene relationship graph G are deleted, perform random sampling operations with nodes $v_j$ and $v_r$ as the starting nodes. For example, taking a node $v_j$ as the starting node, $\gamma$ nodes are randomly selected in the genetic relationship graph *G* and the links with the node $v_j$ are established respectively to form a new edge, the new edge is marked as a negative sample. The set of all negative samples is called the negative sample set *Neg*. Next, Eq. (5) is used to calculate the similarity between any two nodes $v_j$ and $v_r$.

5 $$\begin{aligned} {\text {sim}}\left( v_{j}, v_{r}\right) =\frac{\sum _{\varphi =1}^{\pi } z_{v_{j}}^{\varphi } \times \sum _{\varphi =1}^{\pi } z_{v_{r}}^{\varphi }}{\sqrt{\sum _{\varphi =1}^{w}\left( z_{v_{j}}^{\varphi }\right) ^{2}} \times \sqrt{\sum _{\varphi =1}^{w}\left( z_{v_{r}}^{\varphi }\right) ^{2}}} ,\quad \varphi =1,2, \ldots , \varpi \end{aligned}$$

In the Eq. (5), $z_{v_{j}}^{\varphi }$ represents the value of the feature vector $z_{v_{j}}$ in the $\varphi$-th dimension, and *w* represents the dimension of the feature vector $z_{v_{j}}$. Then use the average similarity of all node pairs in the positive sample set and the average similarity of all node pairs in all negative sample sets to construct the loss function shown in Eq. (6).

6 $$\begin{aligned} L=E_{\left( v_{j}, v_{r}\right) \in {\text {Pos}}}\left[ -\log \left( \sigma \left( {\text {sim}}\left( v_{j}, v_{r}\right) \right) \right) -\sum _{\left( {\bar{v}}_{j}, {\bar{v}}_{r}\right) \in {\text {Neg}}}\log \left( \sigma \left( {\text {sim}}\left( \overline{v_{j}}, \overline{v_{r}}\right) \right) \right) \right] \end{aligned}$$

In the Eq. (6), *L* represents the loss value, *E* represents the averaging operation, $\left( v_{j}, v_{r}\right) \in Pos$ represents the two nodes in the positive sample set *Pos*, ${v_j}$ represents the node selected for the random collection operation with node $v_j$ as the starting node, and ${v_r}$ represents the node $v_r$ is the node selected by the starting node for random sampling operation, and $\left( v_{j}, v_{r}\right) \in Neg$ represents two nodes in the negative sample set *Neg*. Use the random gradient descent method to train the loss function, and calculate the loss value *L* during each training. When the absolute value of the difference between the loss values during two adjacent trainings is less than the given threshold $\delta$, the iteration is stopped.

Finally, Eq. (7) is used to calculate the Mean reciprocal rank(MRR) of the link prediction model generated during each training process, and use the link prediction model with the highest average reciprocal rank as the optimal link prediction model.

7 $$\begin{aligned} M R R=\frac{1}{\varepsilon } \sum _{\tau =1}^{\varepsilon } \frac{1}{{\text {rank}}_{\tau }} \quad \tau =1,2, \ldots , \varepsilon \end{aligned}$$

In the Eq. (7), *MRR* represents the average reciprocal rank, and *rank* represents the rank number of the scores from highest to lowest when the $\varepsilon$-th edge in the positive sample set scores the corresponding $\tau$-th edge in the negative sample set. In the training process, we use the optimal model parameters as the prediction model, and perform link prediction on the graph *G*, generate new edges, and obtain a new gene relationship graph $G^*$.

### Feature selection method based on spectral clustering

After getting the gene relationship graph $G^*$, we can use spectral clustering technology to cluster and select features. Firstly all nodes in the new gene relationship graph $G^*$ are defined as $E=\left( e_{1}, e_{2}, \ldots , e_{\zeta }\right)$, where $\zeta$ represents the total number of nodes in the gene relationship graph $G^*$. Equation (8) is applied to calculate the similarity $w_{\rho 1, \rho 2}$ between any two nodes $e_{\rho 1}, e_{\rho 2}$, $w_{\rho 1, \rho 2}$ is composed into an $\zeta$ -dimensional similarity matrix *W*.

8 $$\begin{aligned} w_{\rho 1, \rho 2}=\sum _{\rho 1=1, \rho 2=1}^{\zeta } \exp \frac{-\left\| e_{\rho 1}-e_{\rho 2}\right\| ^{2}}{2 \Omega ^{2}}, \quad e_{\rho 1}, e_{\rho 2} \in E \end{aligned}$$

where $\Omega$ represents the neighborhood width used to control the node. Next, the sum of all elements in each row of the similarity matrix *W* is calculated to get $d=\left\{ d_{1}, d_{2}, \ldots , d_{\eta }, \ldots d_{\zeta }\right\}$, where $d_\eta$ represents the sum of all elements in the $n-th$ row, The parameter *d* is used to construct a diagonal matrix *D* with dimension $\zeta$, and calculate the Laplacian Matrix $L_{r e y m}=D^{-1 / 2}(D-W) D^{-1 / 2}$ and its eigenvalues. The eigenvalues in ascending order, according to the number $\mu$ of clusters. The first $\mu$ eigenvalues and calculate the corresponding eigenvector $\left\{ \chi _{1}, \chi _{2}, \ldots , \chi _{\mu }\right\}$. $\mu$ eigenvectors $\left\{ \chi _{1}, \chi _{2}, \ldots , \chi _{\mu }\right\}$ are used to form a matrix *U* with $\zeta$ row and $\mu$ column, that is, the matrix $U=\left\{ \chi _{1}, \chi _{2}, \ldots , \chi _{\mu }\right\}$.

Finally, the spectral clustering algorithm is used to cluster the eigenvectors in each row of the matrix *U* to obtain $C=\left\{ C_{1}, C_{2}, \ldots , C_{v}, \ldots , C_{\mu }\right\}$, where $C_{v}$ represents the clusters of the eigenvectors in the *v*-th row. According to the obtained cluster *C*, all the nodes in the new gene relationship graph $G^*$ are divided into $\mu$ groups, and $\mu$ subgraphs are obtained, denoted as $G^{*}=\left[ G_{1}, G_{2}, \ldots , G_{v}, \ldots , G_{\mu }\right] =\left[ \left( v_{1}^{\prime }, \varepsilon _{1}^{\prime }\right) ,\left( v_{2}^{\prime }, \varepsilon _{2}^{\prime }\right) , \ldots ,\left( v_{v}^{\prime }, \varepsilon _{v}^{\prime }\right) , \ldots ,\left( v_{\mu }^{\prime }, \varepsilon _{\mu }^{\prime }\right) \right]$.

To apply the feature selection method for biomarker selection on the clustered subgraphs, we converted the graph structure to a matrix format and used an Embedded feature selection method (linear regression) to feature select the matrix data corresponding to each subgraph to obtain the final feature ranking. The feature with the highest weight corresponding to each subgraph is used as the final biomarker. Our method also supports different feature selection models to evaluate the node weight of each subgraph, which will be described in detail in the experimental section.

### Ethical approval

This study was performed using available datasets, as per my compliance with ethical standards there were no human or animal participants, and therefore, the study did not require ethics approval.

### Research involving human and animal participants

This article does not contain any studies with human participants or animals performed by any of the authors.

## Experimental results and analysis

### The proposed method compared with traditional methods

We compared the proposed method with the traditional feature selection method on two public data sets (DLBCL and Prostate). The dataset details can be found in Table 1.

We first used the David tool for gene ID conversion to obtain gene association information from the GeneMANIA website, used the association information to build graph structure data, and used the gene expression values as the initial state vectors of the nodes with the same dimensionality as the number of samples. The GNN was set to 10 layers in the experiment, and SVM was used as the classifier, and the 5-fold cross-validation average classification accuracy was used as the final result. To find the effect of different number of features on the results, we set the number of clusters (the number of features in the final output) to 1–15, respectively. The results for more number of features and more evaluation metrics are provided in Supplementary Material.

**Table 1:** Data set description and introduction, UR denotes unbalance rate.

Datasets	Samples	Features	Distribution	UR
DLBCL	77	7129	DLBCL:58, FL:19	3.05
Description: DLBCL patients (58) and follicular lymphoma (19)				
Prostate	102	12625	Tumor:52, Normal:50	1.31
Description: Prostate (52) and non-prostate (50)				

**Figure 3:**
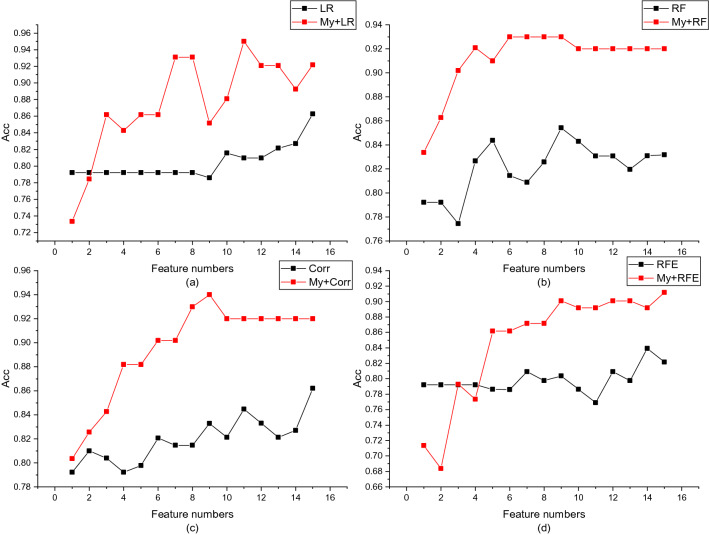
The proposed method is compared with the traditional method in the DLBCL dataset. Figures (**a**) to (**d**) compare logistic regression model (LR), random forest (RF), Pearson correlation coefficient (Corr) and recursive feature elimination method (RFE).

**Figure 4:**
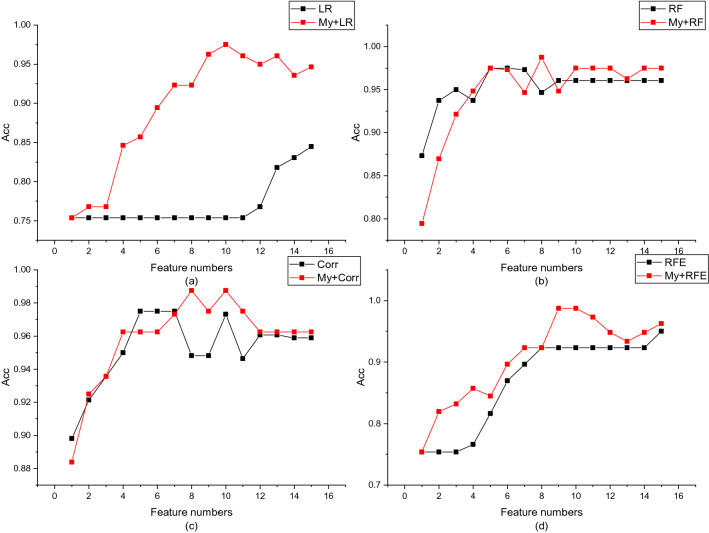
The proposed method is compared with the traditional method in the Prostate dataset. Figures (**a**) to (**d**) compare logistic regression model (LR), random (RF), Pearson correlation coefficient (Corr) and recursive feature elimination method (RFE).

It should be noted that the proposed method defaults to a linear model in the evaluation of sub-cluster nodes, which can be replaced according to the data. It allows a flexible combination of different feature selection methods with the proposed method. In order to prove the effectiveness of the proposed method, in the last step of spectral clustering, we set the sub-cluster feature evaluation method as a contrast method. The experimental results are shown in Figs. 3 and 4. More detailed results and evaluation indicators on figures 3 and 4 can be found in the supplementary files.

It can be seen from Figs. 3 and 4 that the proposed method can significantly improve the feature selection effect and remove redundant features. The proposed methods have good classification accuracy under different numbers. Especially in the linear model, the average classification accuracy of DLBCL and Prostate have been improved by 10.90% and 16.22% respectively. We noticed that in Figs. 3a, d and 4a, the traditional feature selection method continuously adds redundant features, and the classification accuracy is slowly improved, while the method we proposed can significantly remove redundant features and quickly improve classification accuracy.

### Link prediction performance evaluation

**Figure 5:**
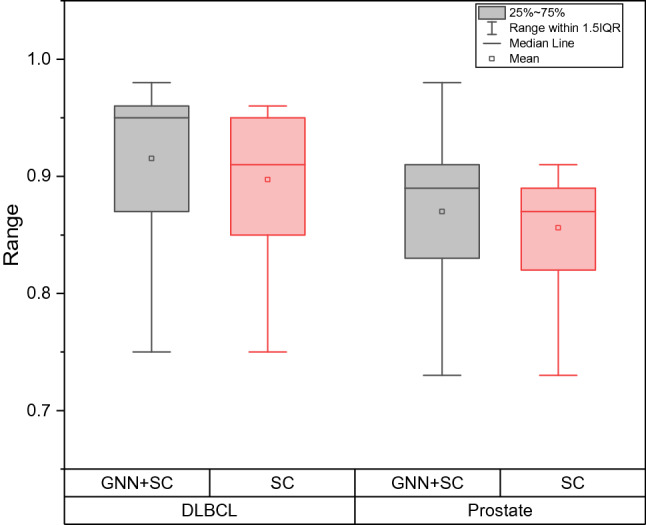
The impact of link prediction on classification results, the fluctuation range of classification accuracy is calculated on the vertical axis.

In this section, we mainly evaluate the gains of the proposed link prediction method for improving the effect. The experiment was performed on DLBCL and Prostate data. We selected the number of features from 1 to 15 respectively and compared the classification accuracy after link prediction with or without graph neural network model. The detailed results are shown in Figure 5.

It can be seen from Figure 5 that after link prediction, the average classification accuracy of the model has been partially improved, and the average classification accuracy of DLBCL and Prostate have been improved by 1.96% and 1.31% ,respectively. Among them, the highest classification accuracy rate on the Prostate dataset has been significantly improved. This shows that the link prediction method we proposed has a significant effect on improving the effect of spectral clustering.

### Comparison with published advanced methods

In this section, we compare the proposed method with the methods used in a variety of published literature on the DLBCL dataset. The detailed results are shown in Table 2. It can be seen from the results that our proposed method is better than the advanced hybrid feature selection method. When the number of features is the same, the classification accuracy is improved by 16.98% compared to SFS-MB^46^.

**Table 2:** Comparison with published advanced methods.

Papers	Method	Feature numbers	Acc
Jinthanasatian et al.^41^	Neuro-fuzzy	13	83.31
Salem et al.^42^	IGGA	110	94.80
Agarwalla et al.^43^	MFDPSO	15	90.01
Wang et al.^44^	IWSSr	15	93.60
Medjahed et al.^45^	BDF	15	89.44
Yang et al.^46^	SFS-MB	15	80.90
Jian et al.^47^	TSVM	15	91.83
Apolloni et al.^48^	BDE-X Rankf	15	92.90
Our method	GNNSC	15	94.64

### Biomarker analysis

**Figure 6:**
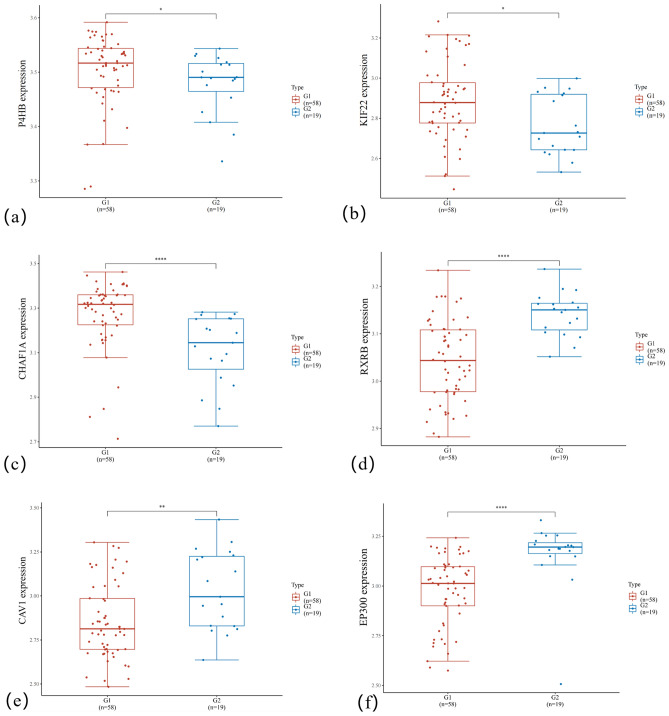
The expression distribution of genes in tissues, where figures (**a**) to (**f**) correspond to different genes, the horizontal axis represents different groups of samples (G1 represents positive samples and G2 represents negative samples), the vertical axis represents the gene expression distribution, where different colors represent different groups. Wilcoxon rank sum test is used here,* represents *p*< 0.05, ** represents *p*< 0.01, **** represents *p*< 0.0001.

In this section, we analyzed the six most important genes selected by our method in the DLBCL data set, these genes were from the top six genes of the GNNSC results. The corresponding probe IDs and gene names are shown in Table 3. In order to analyze the distribution of genes among different samples, we draw the expression distribution of genes on positive and negative samples. The purpose is to observe the difference in gene expression in different groups and to obtain clues about gene function. The expression distribution of the six genes is shown in Figure 6. It can be found that the six genes selected by the proposed method can effectively distinguish the positive and negative samples.

**Table 3:** The six most important genes and their probe IDS selected by the proposed method.

Number	1	2	3	4	5	6
Probe ID	J02783_at	D38751_at	U20979_at	X65463_at	Z18951_at	U01877_at
Gene name	P4HB	KIF22	CHAF1A	RXRB	CAV1	EP300

## Conclusions

This paper proposes a feature selection method based on graph neural network and spectral clustering technology for microarray data analysis. The method effectively uses prior knowledge to construct a gene relationship network and uses graph neural network and link prediction technology to improve feature dependence. Then, it uses spectral clustering technology to cluster redundant features, and uses a linear model to evaluate the features of each subcluster, and output important features. The experimental results show the effectiveness and advancement of the proposed method. Our method can also be combined with different feature selection models to evaluate subcluster features and handle different data flexibly.

In the future research, we will pay more attention to the multiple dependencies in the gene network, and improve the gene relationship network by fusing multiple dependencies. At the same time, we will consider combining the feature selection model with spectral clustering technology for feature selection, rather than applying feature selection after spectral clustering technology.

## Supplementary Information

Supplementary Information 1.

Supplementary Information 2.
