# Supplementary material for: Determination of biomarkers from microarray data using graph neural network and spectral clustering
Source: Sci Rep. 2021 Dec 13;11:23828. doi: 10.1038/s41598-021-03316-6 (PMC8668890; doi:10.1038/s41598-021-03316-6)
Supplement: Supplementary file 1 — Supplementary Information 1. [file 41598_2021_3316_MOESM1_ESM.pdf]

Datasets and GPL files can be downloaded from https://github.com/xwdshiwo/BioFSDatasets.
More detailed results about figure 2 and figure 3 can be found in Excel file. The results include AUC and ACC results under different methods, and the number of features is expanded to 50.
